# Large Language Models for Thematic Summarization in Qualitative Health Care Research: Comparative Analysis of Model and Human Performance

**DOI:** 10.2196/64447

**Published:** 2025-04-04

**Authors:** Arturo Castellanos, Haoqiang Jiang, Paulo Gomes, Debra Vander Meer, Alfred Castillo

**Affiliations:** 1 Mason School of Business, William & Mary, Williamsburg, VA, United States; 2 College of Informatics, Northern Kentucky University, Highland Heights, KY, United States; 3 Information Systems and Business Analytics Department, College of Business, Florida International University, 11200 SW 8th Street, Miami, FL, 33199, United States, 1 305-348-4610

**Keywords:** artificial intelligence, generative AI, large language models, ChatGPT, machine learning, health care

## Abstract

**Background:**

The application of large language models (LLMs) in analyzing expert textual online data is a topic of growing importance in computational linguistics and qualitative research within health care settings.

**Objective:**

The objective of this study was to understand how LLMs can help analyze expert textual data. Topic modeling enables scaling the thematic analysis of content of a large corpus of data, but it still requires interpretation. We investigate the use of LLMs to help researchers scale this interpretation.

**Methods:**

The primary methodological phases of this project were (1) collecting data representing posts to an online nurse forum, as well as cleaning and preprocessing the data; (2) using latent Dirichlet allocation (LDA) to derive topics; (3) using human categorization for topic modeling; and (4) using LLMs to complement and scale the interpretation of thematic analysis. The purpose is to compare the outcomes of human interpretation with those derived from LLMs.

**Results:**

There is substantial agreement (247/310, 80%) between LLM and human interpretation. For two-thirds of the topics, human evaluation and LLMs agree on alignment and convergence of themes. Furthermore, LLM subthemes offer depth of analysis within LDA topics, providing detailed explanations that align with and build upon established human themes. Nonetheless, LLMs identify coherence and complementarity where human evaluation does not.

**Conclusions:**

LLMs enable the automation of the interpretation task in qualitative research. There are challenges in the use of LLMs for evaluation of the resulting themes.

## Introduction

### Background

Qualitative studies in health care shed light on the perceptions, narratives, and discourses that underlie human behavior. This approach enhances understanding of both clinicians and patients’ experiences and expectations, thereby informing decision-making for health policy [1]. Traditionally, these studies involved data collection through face-to-face interviews, observation or artifact analysis, transcription, and manual human coding for sense-making. Recent online advances, such as social media interactions, online reviews, news articles, and in-depth forum discussions, allow researchers and policy makers to collect larger data samples at lower time costs compared with direct interviews [2]. The advent of text mining tools, which allow researchers to cluster text samples into groups based on statistical similarity, has enabled partial automation of the sense-making step. For instance, the use of natural language processing (NLP) to identify risk factors from unstructured free-text clinical notes [3]. Yet, these tools provide only the groupings, leaving the human to apply thematic interpretation [4,5].

Recent advances in generative artificial intelligence (AI) provide valuable tools for researchers conducting qualitative studies, offering support in both data analysis and interpretation. In particular, large language models (LLMs), which are statistical models built using internet-scale datasets, can generate human-style writing in response to natural-language prompts, and assist in analyzing textual data to identify patterns, themes, and underlying meanings [6]. LLMs can aid researchers in conducting thematic analysis by identifying recurrent themes, concepts, or ideas across a dataset supporting the automation of thematic interpretation.

### Previous Work

Topic modeling is a popular approach to uncovering insights in text mining. It identifies patterns in word usage and clusters words into topics, making it a popular method for exploring large, unstructured text datasets. Latent Dirichlet allocation (LDA) is a widely applied method for topic modeling. Previous work has used LDA modeling to analyze social media data and derive insights on key topics [4,7,8]. Despite the new perspectives LDA approaches offer for scientific research [9], using LDA for topic modeling presents challenges [10], notably the significant role of human interpretative contribution in the process [11], which limits scalability. In addition, there is a noted lack of user-friendly tools that support the entire workflow, necessitating a human-in-the-loop to interpret the derived topics. In this paper, we argue that LLMs can help resolve some of the challenges of LDA analysis, specifically in interpreting and labeling topics.

LLMs are emerging as an increasingly reliable and effective tool for interpretative qualitative research, combining the scale that computational techniques allow for with the human’s qualitative logic [12,13]. Previous studies show that ChatGPT (OpenAI) yields comparable results to manual coding with substantial time savings [14]. These studies compare emergent themes in human and AI-generated qualitative analyses, revealing similarities and differences. For instance, some themes are recognized by human coders but missed by ChatGPT, and vice versa [15]. LLMs can highlight novel connections within the data that are not apparent to human coders. In both deductive and inductive thematic analysis, ChatGPT extended the researchers’ views of the themes present in the data [12].

There are challenges associated with the use of LLMs. In the previously cited study [14], ChatGPT was able to recreate the themes originally identified through more traditional methods. However, it was less successful at identifying subtle, interpretive themes, and more successful with concrete, descriptive themes. LLMs may miss themes that require a deep understanding of context or specific domain knowledge. For example, themes related to niche cultural practices or specific professional areas may not be accurately identified by AI without targeted training.

LLMs can also reflect biases present in its training data, potentially overlooking or misinterpreting themes that deviate from its learned patterns. On the other hand, LLM analyses can identify patterns and themes that might be overlooked by human coders due to their preconceived notions or cognitive biases. Further challenges associated with the use of LLMs are shown in Table 1.

**Table 1.** Challenges of large language models.

Challenge	Description	Citations
Ambiguity resolution	LLMs^a^ might struggle to disambiguate certain terms or topics, leading to unclear topic categorization.	[16,17]
Overfitting	LLMs can sometimes focus too much on common or popular topics, missing out on niche or less frequently discussed topics.	[18,19]
Lack of context	Without external knowledge or the ability to track long-term context, LLMs might misinterpret or miss certain topic nuances.	[20]
Bias	LLMs are trained on vast amounts of data, which may contain biases. This can affect topic analysis results.	[21,22]
Overgeneralization	LLMs might overly generalize topics, missing out on specific subtopics or nuances.	[23]
Sensitivity to input	Small changes in input phrasing can sometimes lead to different topic interpretations by the LLM.	[24]
Memory limitations	Due to token limits, LLMs might not capture very long or detailed discussions effectively for topic analysis.	[25]
Interactivity limitations	While LLMs can process static text effectively, they might struggle with dynamic topic analysis, where user feedback or real-time adjustments are required.	[26]

a LLM: large language model.

Given these challenges, some studies suggest that the most effective qualitative analyses may involve a combination of human and AI insights, as human coders often recognize nuanced themes related to context, emotions, and cultural subtleties that AI may miss. For example, a study demonstrates the feasibility of AI as a research assistant, presenting a successful case-study of human-AI collaboration in research by merging the efficiency and accuracy of ChatGPT with human flexibility and context awareness [27]. In addition, the usefulness of ChatGPT in qualitative analysis may depend on the researcher’s ability to ask appropriate questions (prompts), with the output evaluated and supplemented by a human researcher before the final report and publication [28].

There is little guidance in the literature about how LLMs can be integrated into thematic analysis. Challenges associated with the use of LLMs, including overgeneralization and overfitting, need to be investigated in the context of using LLMs for interpreting the relevance of identified topics. Our focus in this work considers inductive thematic analysis, where themes are derived from data without preconceived frameworks, and semantic analysis, in which themes are identified within the explicit content of the data [29]. We plan to consider a hybrid inductive and deductive approach in future work [30].

### Study Objectives

This study considers the possibility of enhancing human productivity by applying LLMs in the interpretation and labeling stage of topic modeling. We present a case study in which data were gathered from an online forum and grouped using text mining tools, and then interpreted for themes in parallel: (1) by human coders and (2) by providing text samples from each classification group to an LLM and prompting the LLM for thematic summarization.

We compared the human- and LLM-generated themes along 4 qualitative dimensions: alignment, convergence, coherence, and complementarity. Based on this analysis, we demonstrate the feasibility of using an LLM to support human thematic interpretation for qualitative research and offer insights into where researchers may find benefit in using LLMs to support thematic interpretation, and where they should exercise caution.

## Methods

### Overview

The proposed methodology is based on three phases: (1) construction of a dataset and topic modeling using LDA, (2) labeling identified groups into topics through human interpretation and through use of LLM, and (3) comparison of identified topics.

### Data Collection and Preprocessing

The data comprises discussions from a publicly accessible Nurse Forum [4]. Data come from posts aggregated over 28 2-week periods from March 2020 to April 2021. Our preprocessing approach ensures that the data is clean, standardized, and focused on the most relevant linguistic features, allowing for a clearer identification of the key aspects discussed in the nurse forum over time. Texts were tokenized using the Python library Gensim [31]. Preprocessing included lowercasing and removing punctuation to ensure uniformity and reduce noise in the text. Stop words, including domain-specific terms like “covid” and “covid 19,” were removed, in addition to those in the Natural Language Toolkit (NLTK) library, to focus on meaningful content. Bigram and trigrams were added to the corpora to identify common multiword expressions, which enhances the detection of contextually significant phrases. Finally, texts were lemmatized using SpaCy (Explosion) [32], retaining only nouns, adjectives, verbs, and adverbs, to normalize words to their base forms and reduce dimensionality.

### Topic Modeling

Topic modeling was conducted using LDA to identify underlying themes in the text data. The LDA algorithm began with random assignments of topics to documents and words to topics. Through iterative optimization, it adjusts these assignments based on the likelihood of word-topic and topic-document distributions. We experimented with different numbers of topics and adjusted hyperparameters, to find the optimal model configuration. Coherence scores, which measure the semantic similarity of words within a topic, were computed for each run. Higher coherence scores indicate more meaningful and interpretable topics. The model with the highest coherence score was selected [33].

This optimal model is then used to extract the top keywords for each topic, summarizing the themes present in the data. The distribution of topics across the corpus was visualized to interpret their prevalence in individual documents and the entire dataset, providing insights into the prominent themes discussed in the nurse forum during the specified period.

### Identification of Topics Through Human Interpretation

Thematic analysis was conducted by 2 coders working independently to familiarize themselves with the data by exhaustively reading the top 10 posts within each topic (ranked based on coherence scores) generated by the topic models [34]. The selected theme names for the labeled topics were compared, which achieved an initial interannotator agreement of 68% (210/310), and 94% (292/310) after a subsequent round. For the remaining 6% (18/310), the underlying posts were examined together to resolve the disagreements, which left no unresolved annotations. The interpretation analysis resulted in 16.5% (15/310) of the identified themes being categorized as having low coherence.

### Theme Derivation Using Large Language Models

Following topic modeling, an LLM was used to derive themes from the identified topics. We created a custom function that takes a system message and a list of user-assistant message pairs, ensuring proper formatting and role assignment. We use the GPT-3.5 based model, specifying the structured messages, temperature, and seed for reproducibility [35]. The system prompt is embedded ensuring consistency in use of the associated set of instructions. The model was chosen for its advanced NLP capabilities, including context-awareness and adaptability to specific thematic contexts, and accessibility to the research team. The prompt instructs ChatGPT to generate themes and subthemes for more nuanced theme identification, addressing the issue of overly broad categorizations observed in initial experiments. An overview of the modeling steps is provided in Multimedia Appendix 1.

### Comparison of Identified Topics

The reliability of coding textual data can be challenging as the goal in content analysis is to attain a “scientific” analysis characterized by reliability, which implies stability in the phenomenon being studied and explicit analytic procedures to ensure that any reasonably qualified person would yield identical results [36]. Intercoder agreement emerges as a key tool in achieving a reliable coding scheme, assessing the extent to which coders assign identical codes to the same set of data [34]. A 5-item ordinal scale typically measures this agreement, with the anchors of “Perfect Agreement,” representing where coders completely agree on codes or categories assigned to data, and “Slight Agreement,” representing very little consensus, or significant disagreement, among the coders in how they code the data. This agreement scale is provided in Multimedia Appendix 1.

A novel 7-point scale was developed following a pilot test conducted by two of the authors to address the complexities of comparing codes generated by humans and ChatGPT. This scale, presented in the first column of Table 2, focuses on exploring the complementary and divergent insights between human-generated and ChatGPT-generated codes. It emphasizes the value of examining differences, especially in cases of low coherence among human-coded data, which allows researchers to uncover nuanced perspectives and understandings contained in ChatGPT-generated themes and within subthemes variability, with the possibility of revealing new and meaningful insights. It serves as a dynamic tool that stresses the importance of learning from intercoding differences rather than seeking strict agreement and validation, as is valued among qualitative researchers [37].

**Table 2.** Agreement between large language model (LLM) and human coding.

Agreement scale	Number of topics, n	Rate of agreement, %
ChatGPT and human coding themes are aligned, coders largely interpret and code the data in a consistent manner.	95	30.6
Substantial agreement: ChatGPT’s subthemes are aligned with human coding, some subthemes provide complementary perspectives or unique insights.	101	32.6
Substantial agreement: ChatGPT’s themes are divergent, human coding classified as low coherence.	51	16.5
Moderate agreement: there is a reasonable level of consensus between ChatGPT and human coding, but there are significant differences in interpretation or coding for some subthemes.	15	0
Fair agreement: ChatGPT’s themes are considered too broad, there are substantial discrepancies between ChatGPT’s subthemes compared with human coding.	30	0
Poor agreement: ChatGPT’s theme specific, yet divergent from human coding.	4	0
Poor agreement: ChatGPT’s theme specific, yet low coherence in human coding.	14	0
Grand total	310	79.7

We then use GPT-4 for topic comparison, accessing the ChatGPT engine through an application programming interface (API) for programmatic purpose. Each prompt included the human-coded themes and the LLM-generated themes, requesting the LLM to assess the agreement based on 4 criteria: alignment, convergence, coherence, and complementarity between the themes. A detailed overview of the prompts is provided in Multimedia Appendix 1.

Alignment assesses the correspondence between ChatGPT and human themes in terms of contextual agreement between the themes [38], rather than lexical agreement. Convergence provides a similar comparison at the level of specific “ChatGPT Subthemes” with reference to the “Human Theme.” Coherence evaluates the logical consistency within the “ChatGPT Theme” and its subthemes, emphasizing the cohesion in both logic and meaning [39]. Complementarity looks at whether the ChatGPT subthemes offer valuable additional insights or perspectives that enhance the human theme by providing detailed mechanistic explanations that align with and build upon the established human theme without contradicting it [40,41],

LLM outputs were parsed to extract values for alignment, coherence, convergence, and complementarity. Human coders then compared the remaining results of reliability analysis with the LLM-generated comparison.

### Ethical Considerations

This study does not involve human subjects, identifiable private information, or direct interactions with individuals. Instead, it relies exclusively on publicly available, anonymized social media posts. Consequently, institutional review board approval was deemed unnecessary.

## Results

### Analysis of Reliability

The LDA analysis identified 310 topics. In thematic analysis, the team considered the topics identified, groups of words, and representative blog post samples in each topic and categorized the 310 topics into 58 subthemes.

A total of 2 authors independently classified the level of agreement on each topic against the themes and subthemes generated by ChatGPT, using the 7-point agreement scale in Table 2. The authors then met to compare assessments and resolve disagreements. The overall reliability is estimated at 79.7% (247/310), which represents substantial agreement according to the intercoder reliability benchmark [36].

Table 2 provides a breakdown of agreement along the comparison scale, with 30.6% (95/310) reflecting taxonomic agreement in themes identified by the human coder and ChatGPT. For example, in one case the human-coder’s theme is “PPE resource availability and control” and the ChatGPT theme is “Mask Availability and Usage in Healthcare Settings.”

In 32.6% (101/310) of the themes the agreement is at the subtheme level. For example, in one instance the human-coded theme is “Testing policies in different settings,” while the ChatGPT theme is “Challenges and Controversies Surrounding COVID-19 Testing,” which was not considered at the same level of specificity of the human coder’s theme. The ChatGPT subthemes are “Allocation of Testing Resources,” “Flaws in Testing Systems,” and “Impact on Public Health and Society.” In the first subtheme the discussion revolves around whether COVID-19 tests should be prioritized for hospitalized patients or health care workers, matching the theme identified by humans. Adding to the reliability of the method, we have the agreement on the lack of coherence of the posts included in the LLM topic, representing 16.5% (51/310) of the topics.

### Alignment and Convergence

LLM provided results on alignment and convergence that we compare with the human evaluation of agreement. The results are displayed in Table 3.

**Table 3.** Analysis of alignment (theme level) and convergence (subtheme level).

		Alignment: Compare the “human theme” and the “ChatGPT theme”			Convergence: Compare the specifics in “ChatGPT Subtheme” with the “human theme.”		
Agreement scale	Total, n	Aligned, n	Misaligned, n	Meets expectation, %	Convergent, n	Divergent, n	Meets expectation, %
ChatGPT and human coding themes are aligned, coders largely interpret and code the data in a consistent manner.	95	86^a^	9	91	86^b^	9	91
Substantial agreement: ChatGPT’s subthemes are aligned with human coding, some subthemes provide complementary perspectives or unique insights.	101	90^a^	11	89	91^b^	10	90
Substantial agreement: ChatGPT’s themes are divergent, human coding classified as low coherence.	51	6	45^c^	88	5	36^d^	71
Moderate agreement: there is a reasonable level of consensus between ChatGPT and human coding, but there are significant differences in interpretation or coding for some subthemes.	15	10^a^	5	67	11^b^	4	73
Fair agreement: ChatGPT’s themes are considered too broad, there are substantial discrepancies between ChatGPT subthemes compared with human coding.	30	18	12	0	17	13^d^	43
Poor agreement: ChatGPT’s theme specific, yet divergent from human coding.	4	1	3^c^	75	1	2	0
Poor agreement: ChatGPT’s theme specific, yet low coherence in human coding.	14	2	12^c^	86	1	8^d^	57

a Expectation is “aligned” for items in the agreement scale.

b Expectation is “convergent” in the agreement scale.

c Expectation is “misaligned” in the agreement scale.

d Expectation is “divergent” in the agreement scale.

We found high level of alignment and convergence for themes classified as high on agreement by human coder. For scale item 1 there was 91% (86/95) alignment and 91% (86/95) convergence, and for scale item 2, there was 89% (90/101) alignment and 90% (91/101) convergence. As expected, we find misalignment for scale items 3 and 7.

The results for scale item 5 (ChatGPT’s themes are considered too broad, there are substantial discrepancies between ChatGPT subthemes compared with human coding) reveal specific nuances of the LLM comparison. Although we expect subthemes to be divergent based on human classification, only 43% (13/30) were classified as divergent by the LLM. For example, a topic labeled by human-coders as “Knowledge about virus,” due to posts in general discuss the nature of COVID-19, was labeled by LLM as “COVID-19 and its implications for healthcare workers,” which is considered much broader although aligned. However, the first subtheme, “Understanding the nature of coronaviruses and COVID-19” is both aligned and convergent with human-generated theme while the other two subthemes, “Importance of proper PPE and testing for healthcare workers” and “Concerns and challenges in healthcare settings and home care,” are clearly divergent from the narrow scope defined by human-theme. Although some subthemes may be tangential, the LLM still classifies them as convergent within a broader framework of idea similarity.

### Coherence

Coherence evaluates the logical consistency within the “ChatGPT Theme” and its subthemes. The results are displayed in Table 4. Coherence was high for items 1,2, and 4 in the agreement scale, meeting expectations. We expected coherence to be low for scale item 5. However, contrary to our expectations, ChatGPT identified 97% (29/30) of cases as coherent. Although human interpretation viewed the LLM theme as broad and the subthemes as tangential, the LLM found logical consistency among these items within the broader scope of the theme.

**Table 4.** Analysis of coherence and complementarity.

	Analysis of coherence			Analysis of complementarity	
	Coherent, n	Low coherence, n	Meets expectation, %	Complementary, n	Meets expectation, %
ChatGPT and human coding themes are aligned, coders largely interpret and code the data in a consistent manner.	94^a^	1	99	93^b^	98
Substantial agreement: ChatGPT’s subthemes are aligned with human coding, some subthemes provide complementary perspectives or unique insights.	101^a^	0	100	97^b^	96
Substantial agreement: ChatGPT’s themes are divergent, human coding classified as low coherence.	49	2^c^	4	8	0
Moderate agreement: there is a reasonable level of consensus between ChatGPT and human coding, but there are significant differences in interpretation or coding for some subthemes.	15^a^	0	100	15^b^	100
Fair agreement: ChatGPT’s themes are considered too broad, there are substantial discrepancies between ChatGPT subthemes compared with human coding.	29	1^c^	3	26	87
Poor agreement: ChatGPT’s theme specific, yet divergent from human coding	3	1	0	1	0
Poor agreement: ChatGPT’s theme specific, yet low coherence in human coding	14	0	0	4	0

a Expectation is “coherent” in the agreement scale.

b Expectation is “complementary” in the agreement scale.

c Expectation is “low coherence” in the agreement scale.

Another unexpected result concerns scale item 3, where 96% (49/51) of the topics were marked as coherent despite being rated as “low coherence” by human coders. Contrary to expectations, 49 out of 51 cases were classified as coherent. LLM relies on single posts to generate subthemes with logical consistency. We illustrate this finding with 2 examples.

One topic that ChatGPT themed as “Nurses’ Safety and Well-being” with the subthemes of “Personal sacrifices and concerns for personal safety,” and “Need for better protection and compensation.” However, the second subtheme was generated based on a single post that mentions hazard pay: “It would be nice if hospitals offered hazard pay, but I’m sure they’re also hurting financially given all of the new measures they’re having to put into place. […]; many are losing a lot of anticipated revenue because they’ve canceled their non-emergency surgeries.” There is insufficient evidence to support the inclusion of this theme.

Another topic ChatGPT themed as “Challenges and Considerations in Nursing and Healthcare” with the subthemes of “Trust and Distrust in Healthcare” and “Disparities in Healthcare.” Although these are considered consistent with theme, the first subtheme is based on a post highlighting the impact of past negative experiences on trust, and the second subtheme is described by ChatGPT as emphasizing the importance of recognizing and addressing disparities that affect various groups, such as gender, age, ethnicity, and socioeconomic status; however, it is based on the following post: “There are disparities... People we love and care about. Yes, I think it’s important to identify areas that are of particular concern and groups that are especially vulnerable. We need to learn and use that knowledge to try to improve our collective future.”

A total of 2 topics were classified as low coherence, which agreed with the corresponding “low coherence” human theme designation. ChatGPT themed 1 topic as “Medications and Health Concerns” with the subthemes of “Medication Switch and COVID-19,” “Casual Conversations and Expressions,” and “Concern and Well-Wishes for Health,” yet recognized as low coherence. The second topic, ChatGPT themed as “Controversial Issues in Healthcare” and has subthemes of “Use of Hydroxychloroquine for COVID-19 Treatment,” “Systemic Racism and Police Brutality,” and “Challenges in Ensuring Compliance with Infection Control Measures.”

### Complementarity

The analysis of complementarity is also provided in Table 4. For scale items 1, 2, and 4 the expectation was that the subthemes provide complementarity to the human-generated theme and the results meet expectations (98% (93/94), 96% (97/101), and 100% (15/15), respectively). For example, one topic with the human-generated theme of “Testing policies in different settings” was associated with the ChatGPT subthemes of “Allocation of Testing Resources,” “Flaws in Testing Systems,” and “Impact on Public Health and Society.” The first subtheme is about whether testing availability should be prioritized for hospitalized patients or health care workers, but the second subtheme highlights significant complementary issues with regards to flaws in the CDC’s COVID-19 testing protocols, delays in fixing the tests, and the impact on the ability to detect and track the spread of the virus. The third theme expanded further into the social implications of the impact of inadequate testing resources, limited testing on the perception of the virus’s severity, and the potential spread of the virus due to lack of testing and preventive measures.

Conversely, the expectation for the agreement scale item 5 was that complementarity would be low, yet ChatGPT found 87% complementarity. For instance, in the example mentioned above, the topic labeled by human-coders as “Knowledge about virus,” the subthemes are considered divergent (“Importance of proper PPE and testing for healthcare workers”) and too broad (“Concerns and challenges in healthcare settings and home care”) when compared with the scope defined by “knowledge about the virus.” The posts on these themes cover diverse topics such as the importance of proper personal protective equipment (PPE), concerns about testing and returning to work, the potential risks involved in home care, questions about Health Insurance Portability and Accountability Act (HIPAA) regulations, and the need for research on treatment options. The complementarity of themes only exists in a very broad sense and can be considered as “out of context.”

## Discussion

### Principal Findings

Our study offers several significant insights into the use of ChatGPT for the augmentation of topic models. A key finding is the importance of considering different levels of abstraction in theme analysis. The division into themes and subthemes is crucial for uncovering specific nuances, addressing the risk of overgeneralization inherent in LLMs.

Furthermore, our exploration of subthemes reveals that LLMs, in general, can resolve ambiguity, aiding in the clear categorization of topics, even from a limited dataset. The effective handling of “low-coherence” topics such as “health disparities” and the complementary insights provided on subthemes of “Testing policies in different settings” demonstrate the LLM’s proficiency in navigating and categorizing complex subject matter at the subtheme level.

In terms of overall reliability, our study estimates a 79.7% (247/310) agreement level, positioning it at the high end of substantial agreement (60%-80%) and the low end of almost perfect agreement (80%-100%) on the intercoder reliability benchmark scale. This suggests a robust level of agreement between human coders and the LLM, indicating a reliable consistency in the classification of topics.

However, the examination of alignment and convergence reveals a nuanced aspect of LLM performance. While LLMs exhibit high accuracy in identifying alignment and convergence for topics classified by human analysis as aligned, a notable challenge arises when classifying divergent subthemes. The LLM tends to classify divergent subthemes as convergent, particularly when one of the subthemes converges in similar ideas, leading to a potential misrepresentation of thematic divergence.

The evaluation of coherence, yields an unexpected result, highlighting the issue of “overfitting.” Specifically, topics classified as coherent by the LLM contradict human coders’ assessments of low coherence. This suggests a potential challenge where ChatGPT may force-fit solutions that match specific data points (posts) but are “too good to be true” from a pattern standpoint, lacking the broader pattern consistency expected in thematic coherence. ChatGPT may be construing the theme based on the wealth of data at its disposal.

The analysis of complementarity confirms that LLMs identify subthemes that provide additional insights to themes in human researchers’ findings. LLMs can successfully identify niche topics, showcasing their potential to uncover unique thematic elements.

Our study emphasizes the critical importance of providing adequate contextual framing to ChatGPT-based classification. The challenge of lack of context becomes apparent, as LLMs may misinterpret or overlook certain topic nuances without external knowledge or the ability to track long-term context.

### Limitations

The study is limited by (1) our focus on a single social media source and (2) the LLM used. First, we focus on data from a single nurse forum, but future inclusion of additional social media sites, including those used in other countries and by users who speak other languages, may enhance the results reported here. Furthermore, while we used the OpenAI chat completion API (GPT-3.5 and GPT-4) for thematic analysis due to its accessibility to the research team, other language models have since emerged. These newer models should be tested to determine if they perform better in different contexts. Furthermore, we kept the LLM prompts as simple as possible to demonstrate that even using a simple approach the generative AI could produce solid results. Further work can apply fine tuning to prompting and design approaches to enhance the thematic analysis capabilities of LLMs, such as retrieval-augmented generation (RAG). Finally, we focus on inductive thematic analysis and short form content data. We recognize that long-form text data may pose distinct challenges in applying LLMs.

### Implications

For the LLM challenges found in this study, such as overgeneralization and overfitting, future study may apply different guardrails, such as implement algorithms that detect and mitigate biases during both training and generation phases. These guardrails monitor and filter the outputs of LLMs addressing different requirements such as hallucinations in LLM outputs [42].

Future research could investigate the potential of feeding raw transcripts into ChatGPT and incorporating AI-generated themes into triangulation discussions. By contributing to triangulation, this approach promises to unveil potential oversights, present alternative perspectives, and highlight inherent researchers’ personal biases. By seamlessly incorporating AI into the discourse analysis process, researchers may uncover a richer understanding of the subject matter, fostering a more comprehensive and nuanced exploration of diverse perspectives. This integration not only enhances the depth of analysis but also provides a valuable tool for refining methodologies and mitigating potential biases, ultimately contributing to the advancement of research methodologies in the burgeoning field of AI-driven discourse analysis.

### Conclusions

Overall, this study underscores the multifaceted nature of using ChatGPT for thematic analysis, acknowledging both its strengths and challenges. The insights gained contribute to a more nuanced understanding of the capabilities and limitations of LLMs in handling complex topical data in the healthcare field, offering valuable considerations for future research in the intersection of artificial intelligence and discourse analysis.

## Supplementary material

10.2196/64447 Multimedia Appendix 1 Large language model’s use for thematic analysis and classifying agreements.

## Abbreviations

AI: artificial intelligence
API: application programming interface
HIPAA: Health Insurance Portability and Accountability Act
LDA: latent Dirichlet allocation
LLM: large language model
NLP: natural language processing
NLTK: Natural Language Toolkit
PPE: personal protective equipment
RAG: retrieval-augmented generation
