# Supplementary material for: Large Language Models for Thematic Summarization in Qualitative Health Care Research: Comparative Analysis of Model and Human Performance
Source: JMIR AI. 2025 Apr 4;4:e64447. doi: 10.2196/64447 (PMC12231516; doi:10.2196/64447)
Supplement: Multimedia Appendix 1 [file ai-v4-e64447-s001.docx]

**LLM use for thematic analysis**

We employed a GPT-4 based model (**gpt-4-1106-preview**) for our analysis. The model was chosen for its advanced natural language processing capabilities, including context-awareness and adaptability to specific thematic contexts.

**Detailed overview of the steps**

| **Step** | **Description** | **Example Instance** |
| --- | --- | --- |
| 1. Data Preparation | A row is selected from the primary data DataFrame (**df**). | Row data includes: Coder assessments, Human-derived themes, and ChatGPT-derived themes and subthemes. |
| 2. Prompt Construction | A prompt is constructed for the LLM based on the selected row's data. | Prompt includes: "Coder 1: [coder1 data]. Coder 2: [coder2 data]. Derived theme from coders (human theme): [HumanTheme]. ChatGPT derived theme (ChatGPTTheme): [ChatGPTTheme]. ChatGPT Sub-theme: [ChatGPTSubtheme]." |
| 3. LLM Interaction | The prompt is sent to the LLM using the **chat** function. | The **chat** function processes the prompt and gets the LLM's response. |
| 4. Output Parsing | The LLM's response is parsed using the **parse_output** function. | The response is categorized into Alignment, Coherence, Convergence, and Complementarity based on the content. |
| 5. Result Storage | The parsed results are stored back in the DataFrame. | The DataFrame (**df**) is updated with values like "Aligned", "Coherent", "Convergent", "Complementary" for the respective row. |
| 6. Error Handling | If an error occurs, the row index is logged for tracking. | In case of an error during processing, the index of the row is added to the list for error_rows. |
| 7. Exporting Results | The combined data is exported to a new CSV for analysis. | The final combined DataFrame is saved as **output_1108_success.csv**. |

**Agreement scale**

The classification of coder agreement considered five levels derived from the guidelines established by Landis and Koch in their 1977 paper titled *The Measurement of Observer Agreement for Categorical Data* as further used in [36]:

Almost Perfect Agreement: This represents a scenario where all coders completely agree on the codes or categories assigned to each piece of data. There is no disagreement among the raters.

Substantial Agreement: Substantial agreement indicates that there is a high degree of consensus among coders, but there may still be some minor discrepancies. It suggests that the coders largely interpret and code the data in a consistent manner.

Moderate Agreement: Moderate agreement implies that there is a reasonable level of consensus among coders, but there are significant differences in interpretation or coding for some portions of the data.

Fair Agreement: Fair agreement suggests that there is some level of agreement among coders, but there are substantial discrepancies in how they interpret and code the data. This level of agreement may require further clarification or refinement of the coding process.

Slight Agreement: Slight agreement indicates that there is very little consensus among coders, and there are significant disagreements in how they code the data. This level of agreement may be unacceptable in many qualitative research studies.

**Use the LLM to classify the agreement**

We use the *gpt-4-1106-preview* model to compare human-coded themes derived from the topic analysis with LLM-generated themes.

The *chat* function in our script combines these *system message* and *user_assistant* list to create a conversational context for the LLM. The system message provides guidelines or context for the LLM, directing it on how to analyze the data. The *user assistant list* is constructed for each row in the DataFrame. It includes the prompt, which is a combination of coder assessments, human-derived themes, and ChatGPT-derived themes and subthemes. It instructs the LLM to assess alignment, coherence, convergence, and complementarity between the human-generated and ChatGPT-generated themes. The *parse_output* function in the script is designed to parse and categorize the output received from the Large Language Model (LLM) based on specific thematic dimensions. The function processes this output text and categorizes it into four thematic dimensions: Alignment, Coherence, Convergence, and Complementarity. The *Alignment* dimension assesses how well the "ChatGPT Theme" aligns with the "Human Theme." Alignment doesn't require exact similarity but focuses on contextual agreement between the themes. The output categorizes the theme as either "Aligned" or "Misaligned," with accompanying reasoning determines whether the "ChatGPT Theme" is contextually aligned with the "Human Theme." The *Coherence* dimension examines the logical consistency within the "ChatGPT Theme" and its subthemes. The theme is classified as either "Coherent" or "Low Coherence," reflecting how well the subthemes logically fit together. The *Convergence* dimension compares the specifics in the "ChatGPT Subtheme" with the general "Human Theme." It assesses whether the ChatGPT Subtheme converges (aligns) or diverges (differs) from the Human Theme. The result is marked as "Convergent," "Divergent," or "-" (not clear or not applicable). The *Complementarity* dimension looks at whether the "ChatGPT Subtheme" offers additional insights or perspectives that enhance the "Human Theme" without contradicting it. If the ChatGPT Subtheme adds value to the Human Theme without negating it, it’s marked as "Complementary." If it contradicts the Human Theme, it's labeled as "Contradictory"; otherwise, it's marked as "-".

For each row in the primary DataFrame (df) we construct a prompt for the LLM using data from the row (coder assessments, themes), Send the prompt to the LLM using the chat function, parse the LLM's response using the parse_output function, Store the parsed results (Alignment, Coherence, etc.) in the DataFrame, if an error occurs, log the row index in a list for error tracking

An example of the prompt is provided below.

*Coder 1: "". Coder 2: Sharing Covid symptoms. Derived theme from coders (human theme): Covid effects. ChatGPT derived theme (ChatGPTTheme): Nursing Profession and Working Conditions. ChatGPT Sub-theme: Stress and Challenges of Nursing Jobs; Comparisons with Other Professions; Concerns about Compensation and Safety. I want you to create 4 columns: Alignment, Coherence, Convergence, and complementarity.*

*These are the definitions: Alignment: Compare the "Human Theme" and the "ChatGPT Theme" to determine alignment, it does not need to be verbatim, but contextually aligned. "Aligned"; otherwise, "Misaligned."*

*Coherence: Examine the "ChatGPT Theme" and "ChatGPTSubtheme" for logical consistency. Note that "ChatGPTSubtheme" can have more than one subtheme. In that case each of the sub-themes in the "ChatGPTSubtheme" would be separated by a semi-colon (;). If they seem coherent, mark as "Coherent"; otherwise, "Low Coherence."*

*Convergence: Compare the specifics in "ChatGPTSubtheme" with the general "Human Theme." If they seem to converge on similar ideas, mark as "Convergent"; if they diverge, mark as "Divergent"; if it's not clear or not applicable, mark as "-".*

*Complementarity: If the "ChatGPTSubtheme" provides additional insights or perspectives that complement the "Human Theme" without contradicting it, mark as "Complementary"; otherwise, if it contradicts it mark as "Contradictory", otherwise just mark it as "-".*

*For the alignment, take a deep breath and consider the Human Theme in the context of coder 1 and coder 2. The format of the output should be Alignment: Aligned or not, and then the reasoning, and then do the same with Coherence, Convergence, and Complementarity. Any other conclusion remarks would go at the end.*
